# Poor knowledge – predictor of nonadherence to universal precautions for blood borne pathogens at first level care facilities in Pakistan

**DOI:** 10.1186/1471-2334-7-81

**Published:** 2007-07-24

**Authors:** Naveed Z Janjua, Mahreen Razaq, Subhash Chandir, Shafquat Rozi, Bushra Mahmood

**Affiliations:** 1 Department of Community Health Sciences, Aga Khan University, Stadium Road Karachi-74800, Pakistan; 2 Department of Epidemiology & International Health, School of Public Health, University of Alabama at Birmingham, Birmingham, AL, USA; 3 Chandka Medical College, Larkana, Pakistan; 4 Department of Health Behavior, School of Public Health, University of Alabama at Birmingham, Birmingham, AL, USA

## Abstract

**Background:**

We conducted an assessment of knowledge about blood borne pathogens (BBP) and use of universal precautions at first level care facilities (FLCF) in two districts of Pakistan.

**Methods:**

We conducted a cross-sectional survey and selected three different types of FLCFs ; public, general practitioners and unqualified practitioners through stratified random sampling technique. At each facility, we interviewed a prescriber, a dispenser, and a housekeeper for knowledge of BBPs transmission and preventive practices, risk perception, and use of universal precautions. We performed multiple linear regression to assess the effect of knowledge score (11 items) on the practice of universal precautions score (4 items- use of gloves, gown, needle recapping, and HBV vaccination).

**Results:**

We interviewed 239 subjects. Most of the participants 128 (53%) were recruited from general practitioners clinics and 166 (69.5%) of them were dispensers. Mean (SD) knowledge score was 3.8 (2.3) with median of 4. MBBS prescribers had the highest knowledge score while the housekeepers had the lowest. Mean universal precautions use score was 2.7 ± 2.1. Knowledge about mode of transmission and the work experience alone, significantly predicted universal precaution use in multiple linear regression model (adR^2 ^= 0.093).

**Conclusion:**

Knowledge about mode of transmission of blood borne pathogens is very low. Use of universal precautions can improve with increase in knowledge.

## Background

Health care workers are at a high risk of needle stick injury (NSI) and blood borne pathogens (BBP)[1]. According to a World Health Organization estimate, in year 2002, sharp injuries resulted in 16,000 hepatitis C virus (HCV), 66,000 hepatitis B virus (HBV) and 1000 human immunodeficiency virus (HIV) infections in health-care workers worldwide[2]. Recapping, disassembly, and inappropriate disposal increase risk of NSI [3-5]. In developing countries, the frequency of these factors gets accentuated with high injection use at health care facilities, most of which are provided with previously used syringes [2,6]. Injection use is very common in Pakistan where 13.6 injections per person are administered each year [7]. More than 50% of these injections are provided with previously used syringes [7]. Reuse of the syringe involves manipulation, including recapping and disassembly, that puts providers at the risk of NSI [6]. Prevalence of HBV and HCV in Pakistan is more than 10% (unpublished data) and unsafe injections transmit most of these infections [6,7]. Hence, risk of NSI and associated infections is higher in Pakistan as compared to those countries that have a low prevalence of HBV and HCV.

In Pakistan, more than 80% of the health care is provided at general practitioners' clinics. Most of these clinics consist of a small, single room structure where consultation, injection administration and drug dispensing is performed [7,8]. On average, a practitioner sees 10–100 patients per day and charges may vary from Rs.10 (15 cents) to Rs. 50 (80 cents) [7,8]. Most of the injection prescriptions and reuse of syringes occur at the clinics of general practitioners (GP)[7,8]. Hence, health care workers at these clinics are at a greater risk of NSI than those working at the secondary or tertiary care hospitals. Sharp waste handling within the clinic and the out-of-clinic disposal of this waste is also unsafe, putting the injection providers, as well as the community, at risk of needle sticks[9]. Most of the time, injection providers (nurses or dispensers) working at clinics are not formally qualified, and they learn injection administration while working with someone who already knows it. During their apprenticeship or job the clinic, they never receive training in infection control and universal precautions. Universal precaution training and practices have been shown to reduce blood and body fluid exposure substantially[10]. Not recapping the needles and disposing them safely into puncture resistance containers alone has shown to reduce NSI by almost 70%[11]. Owing to the unique nature of these clinics, interventions needed for these facilities might be different from those that can work for large secondary or tertiary care hospitals, or state owned enterprises, where funds can be made available for NSI prevention programs. These clinics are small, workforce (practitioner assistants), often does not have formal training and also by law, they are not bound to make arrangements for occupational safety. Universal precautions trainings and practices are low cost solution to reducing risk of sharp injuries and have a high likelihood of being adopted. We conducted an assessment of knowledge about BBPs, risk perception and practice of universal precautions at first level care facilities in two districts of Sindh province, Pakistan. This assessment will provide essential baseline data for developing and testing low cost training interventions in universal precautions.

## Methods

### Study design and setting

We conducted a cross-sectional survey of health-care workers at first level health care facilities in two rural districts (Larkana and Mirpur Khas) in Sindh province of Pakistan during January through September 2004. Health care in these districts is provided by 378 general practitioners (GPs), 128 non MBBS practitioners (dispensers) and 35 public Basic Health Units (BHUs). Most of the GPs have clinics in urban areas or on a highway at the junction of 3–4 villages. Usually a clinic consists of 1–2 rooms, with a table for the practitioner, patients' sitting area, a dispensary and sometimes a bed or a couch. In these clinics, besides a physician, there is a dispenser (physician assistant), who is mostly unqualified and dispenses medication and provides injections. Dispensers, who are or have been a physician assistant at a GP clinic, or are working at a public facility, also practice independently, where they mostly prescribe and dispense medication and provide injections personally but may, at times, be assisted by an assistant. Public BHUs typically have one or two physicians and their assistants, a vaccinator, lady health visitor, and a guard or office helper. Average patient turnover at a GP clinic is 25–75 patients per day; at dispenser clinics 10–30 patients and 25–100 patients at a BHU. Patient mix in these clinics varies by socio-economic status (SES), location, and type of clinic. In remote rural areas, where only one or two practitioners are available, patients seek care from them. Where more choices are available as in towns, patients from a higher SES are more likely to visit general practitioners, while low and lower middle income patients are more likely to seek care from public or unqualified practitioners[8]

### Study population

Our study population included health care workers (HCWs) working at public and private sector first level health-care facilities (FLCF) in the study districts. In this study those workers (physicians, dispensers and housekeepers) who are in direct contact with the patients or with equipments used on patients and are likely to get exposure to blood borne pathogens, were included. We used the following definitions for health care workers stratification into three groups: prescribers, defined as those who provide consultation and prescribe medication to the patients – they could either be qualified MBBS practitioners (GPs) or unqualified non-MBBS practitioners; dispensers, those who dispense medicines and administer injections; and housekeepers, who perform the janitorial work at the health care facilities.

### Subject selection

In Pakistan, there is no registration authority that keeps record of the practitioners in an area; therefore we compiled a list of all general practitioners, non MBBS practitioners and public BHUs. We obtained the list of general practitioners from local Pakistan Medical Association chapter and updated it using a list compiled by a pharmaceutical company. We further validated this list from the drug stores. Since there is no association for the non-MBBS providers and pharmaceuticals usually do not target all the non- MBBS providers, we compiled their list by visiting drug stores in each part of the district. We further validated this list with the physicians in the area and the village elders. To our knowledge this is the most complete list of health care workers that can be obtained for this setting. We obtained a list of public BHUs and dispensaries from the district health offices. Once we had a sampling frame, we selected clinics from each of the three categories in rural and urban areas through stratified simple random sampling technique. On average each clinic has a physician, a dispenser and a housekeeper; thus all our groups were likely to be housed in one clinic. By using this method, the required number of dispensers, housekeepers and physicians was deemed to be equal to the number of clinics.

### Sample size

Sample size calculation was done for estimation of NSI based on the assumption that 74% of the dispensers received at least one injury during the entire duration of their job in Pakistan (unpublished data Choudary FN). With 5% confidence level and 5% bound on error of estimation and after accounting for 10% non-response, sample size was 223 dispensers and hence 223 clinics. Thus, we were able to have 223 health care workers in each category. Based on the number of facilities in each category, we distributed 50% of the sample to GPs' clinics, 35% to non-MBBS and 15% to the public facilities. Sample distribution into three types of facilities was based on proportion of patients seeking care from each type of facility in study area[8].

### Interview and questionnaire

Final year medical students who were trained in interviewing and study procedures, interviewed health care workers at their clinics using a questionnaire that had been pretested and translated into Urdu. We explained the purpose, procedure, and risks and benefits of the study to the respondents and obtained a verbal informed consent. Ethics Review Committee at Aga Khan University reviewed and approved the study.

**Figure 1:**
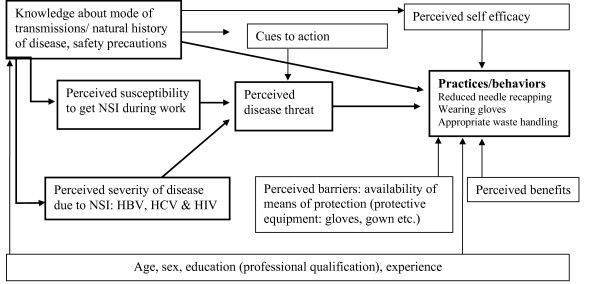
**Conceptual model based on health belief model used in the study **(bold lines depict constructs used in the study)

We used Health Belief Model (HBM) to design our questionnaire[12,13]. In HBM, knowledge influences perception about disease susceptibility and disease severity. Both of these determine perceived disease threat which, in turn, influences behavior. Behavior is also determined by perceived self efficacy (confidence in one's ability to perform certain activity), cues to action, and barriers and benefits (Figure 1). Our questionnaire included information on knowledge, perceived susceptibility, perceived severity and behaviors (Table 1). We measured knowledge about mode of transmission of HBV and HCV using 10 items. One item addressed measurement of knowledge regarding spread of diseases other than HBV and HCV through syringe use "which other pathogens get transmitted with reuse of syringes". We allowed the respondents to spontaneously mention transmission routes. We used open-ended questions to inquire about use of precautions that can lower risk at work place. Perceived susceptibility to acquiring blood borne pathogens was assessed using one item "how much risk of acquiring a BBP is involved in your work", on a scale of 1 (being none) to 5 (being very high). Perceived severity of consequences of needle stick injury was assessed by one item "what can happen if you get a needle stick" with responses of nothing and acquisition of BBP and others. Behaviors (universal precaution practices) included information on vaccination against hepatitis B (yes/no), wearing gloves while performing medical and surgical procedures (measured on a scale of 0 = never to always = 3) and recapping of needle measured as 0 = always to 3 = never. Questionnaire also included information on socio-demographics, professional qualifications and total number of years since start of practice. We also inquired about needle stick injuries during the past six months to one year and circumstances surrounding the latest injury.

**Table 1:** Health belief model constructs used in questionnaire for in study of knowledge about blood born pathogens at first level health facilities in Sindh province of Pakistan 2004

**Items**	**Constructs**
	**Mode of transmissions knowledge (11 items)**
	*Mode of transmissions of hepatitis B and C knowledge(10 items)*
1	Reuse of contaminated syringe (Yes = 1, No = 0)
2	Unscreened blood (Yes = 1, No = 0)
3	Unprotected sexual intercourse (Yes = 1, No = 0)
4	Reuse of razor (Yes = 1, No = 0)
5	Use of unsterilized medical instruments (Yes = 1, No = 0)
6	Needle stick or by injury with sharp object (Yes = 1, No = 0)
7	Exposure to body fluids (Yes = 1, No = 0)
8	If mucosa in oral cavity is ulcerated (Yes = 1, No = 0)
9	Contact with infected person (Yes = 0, No = 1)
10	Sharing combs at home (Yes = 0, No = 1)
11	*HIV transmission through reuse of syringes (Yes = 1, No 0)(1 item)*
	**Perceived susceptibility of acquiring infection at workplace (1 item)**
1	How much risk of acquiring hepatitis B, C and/or HIV is involved in your work setting (1 = None to 5 = Very high)
	**Perceived severity of disease after NSI**
1	What can happen if accidentally any of Health Care Worker got needle stick injury? (1 = infection with any of BBP, 0 = Nothing)
	**Behaviors- universal precautions components (4 items)**
1	Completed HBV vaccination (Yes = 1, No = 0)
2	Wear gloves in procedure where possibility of blood/body fluid exposure (Never = 0 to Always = 4)
3	Wear gown for procedures where possibility of blood/body fluid splash (Never = 0 to Always = 4)

### Statistical analysis

We entered data in Epi-Info version 6.04 and performed analysis using SPSS version 14.0. We computed mean (± SD) for continuous and proportions for categorical variables. We computed the 11 item knowledge score by summing correct responses from 10 items (yes = 1 no = 0) about mode of transmission of HBV/HCV, and transmission of HIV through reuse of syringes, and one item about an infectious agent with the highest likelihood of transmission with reuse of syringe or needle stick injury. We assessed the internal consistency of our knowledge scale using Cronbach's Alpha. We compared knowledge across various demographic variables that can influence knowledge like professional qualification, job title. We assessed the relationship between knowledge and risk perception using Pearson's correlation.

We constructed a score for universal precautions practice from four items; use of gloves, use of gown, recapping of needle after injections, (measured on a scale of 0 = never to always = 3) and vaccination for HBV (yes/no). Recapping was reverse coded before summing so that 0 = always and 3 = never.

We performed multiple linear regression analysis to assess the relationship of modes of transmission knowledge score, precaution knowledge score, perceived risk at work place, perceived severity of disease due to NSI at work place, age, work experience, respondent type and qualification with the practice of universal precautions score. Since age and work experience were highly correlated, we only included work experience. Those variables that were significant at *P *< 0.2 were selected for multivariable model. We assessed the assumptions model fitness using residual plots.

## Results

We were able to interview 239 subjects from 172 clinics. Majority of the participants 166 (69.5%) were dispensers followed by MBBS prescribers 42(17.6%), non-MBBS prescribers 22 (9.6%) and housekeepers 9 (3.8%). Housekeepers were the most difficult group to contact because most of them were not full time employees; they only come early in the morning for cleaning. Physicians were the second category that could not be successfully recruited because either they were busy in patient consultation or were away, in which case, the dispensers opened the clinic and stayed there till late. Thus, we were able to collect information from dispensers in 86% of the visited clinics. The mean (± SD) age of the respondents was 30 ± 10.7 years. On average, health care workers had been working for 9.6 years (Table 2).

**Table 2:** Basic characteristics of health care workers in study of knowledge about blood born pathogens at first level health facilities in Sindh province of Pakistan 2004

**Characteristics**	**n**	**%**
**Age (years) (n = 235)**		
< 20	42	17.9
20–30	78	33.2
30–40	62	25.9
40 or more	53	22.6
Mean age of respondent (Std)	30.0(10.76)	
**District (n = 238)**		
Mirpurkhas	137	57.3
Larkana	101	42.3
**Area (n = 239)**		
Urban	55	23
Rural	184	77
**Facility by owner (n = 239)**		
Public	39	16.3
Private	200	83.7
**Facility by major provider (n = 238)**		
Public physician	29	12.1
GP	128	53.8
Non MBBS	81	33.9
**Respondent type (n = 239)**		
MBBS Prescriber	42	17.6
Non-MBBS prescriber	22	9.2
Dispenser	166	69.5
Housekeeper	9	3.8
**Professional Qualification**		
MBBS	42	17.6
Nursing Diploma	20	8.4
B-Pharmacy	56	23.4
Dispenser/lab technician diploma	12	5.0
Homaopathy	30	12.6
No professional education	79	33.1
**Years of work experience**		
1 st (0–3)	60	25.1
2 nd (4–7)	60	25.1
3 rd (8–14)	66	27.6
4 (> 14)	53	22.18
Mean years of work experience	9.55	7.6

### Knowledge about hepatitis B and C and role of NSI in transmission

Mean (SD) knowledge score computed from 11 items about hepatitis B and C was 3.8 (2.3) with median of 4. The Cronbach's alpha for internal consistency of knowledge items was 0.755. Mean knowledge score of MBBS prescribers (5.7) was highest followed by non-MBBS prescribers (3.7), dispensers (3.4) and housekeepers (1.6, *P *< 0.001). Mean knowledge score of respondents was higher at public (4.0) and GP clinics (4.2) as compared to the non-MBBS practitioner clinics (3.2). MBBS respondents had highest knowledge score (5.7) and those with no professional education had the least (2.7). Mean knowledge score increased with age as well as years of experience (Table 3).

**Table 3:** Mode of transmission of HBV/HCV, precautions at workplace and overall knowledge scores of health care workers at first level care facilities in Sindh Pakistan 2004

		**Mode of Transmission score **^a^			**Precaution scores **^b^			**Cumulative score **^c^		
	**n**	**mean**	**SD**	***P***	**mean**	**SD**	***P***	**mean**	**SD**	***P***

**Respondent type**										
MBBS Prescriber	42	5.71	2.20	< 0.001	1.67	0.78	0.0942	7.40	2.29	< 0.001
Non-MBBS Prescriber	22	3.77	2.47		1.36	0.49		5.14	2.73	
Dispenser	166	3.48	2.05		1.4	0.74		4.93	2.36	
Housekeeper	9	1.56	1.13		1.1	0.33		2.67	1.32	
**Facility by provider**										
Public physician	29	4.03	2.68	0.01	1.52	0.83	0.6695	5.56	2.30	0.013
GP	126	4.17	2.27		1.52	0.75		5.70	2.50	
Non MBBS	83	3.22	2.04		1.43	0.67		4.65	2.39	
**Professional Qualification**										
MBBS	42	5.71	2.18	< 0.001	1.69	0.78	0.3336	7.40	2.29	< 0.001
Nursing Diploma	20	4.45	2.42		1.65	0.81		6.10	2.59	
B-Pharmacy	56	3.95	2.22		1.41	0.63		5.36	2.39	
Dispesing/labtec	12	3.42	1.83		1.42	1.08		4.83	2.21	
Homaopathy	30	3.67	1.55		1.4	0.56		5.06	1.86	
No professional education	79	2.66	1.95		1.44	0.73		4.10	2.39	
**Age (years)**										
< 20	42	2.33	1.86	< 0.001	1.26	0.5	0.0359	3.6	2.16	< 0.001
20–30	78	3.64	2.19		1.49	0.73		5.13	2.53	
30–40	62	4.48	2.07		168	0.78		6.16	2.29	
40 or more	53	4.49	2.47		1.47	0.72		5.96	2.63	
**Work experience quartiles (years)**										
1 st (0–3)	60	2.43	1.78	< 0.001	1.38	0.69	0.432	3.82	2.21	< 0.001
2 nd (4–7)	60	4.15	2.31		1.45	0.67		5.6	2.63	
3 rd (8–14)	66	4.48	2.44		1.55	0.77		6.03	2.67	
4 (> 14)	53	4.15	1.96		1.58	0.77		5.73	2.11	

a: Based on 11 items (mode of transmission)

b: based on 4 items (gloves, gown, recapping and vaccination)

c: Sum of transmission and precaution knowledge score

Majority (39, 92%) of the MBBS prescribers correctly identified reuse of contaminated syringes as a mode of transmission while this proportion was only 15 (35.7%) for needle stick or sharp injury, and 13 (31%) for exposure to body fluids. Similar to MBBS prescribers, 16 (72.7%) non-MBBS prescribers also spontaneously mentioned reuse of syringes while only 4 (18%) and 1 (4.5 %) mentioned needle stick and exposure to body fluids respectively. Among dispensers, 117(72%) mentioned reuse of syringes as a mode of transmission while only 20(12%) mentioned needle stick. Reuse of syringes was the only mode of transmission housekeepers 2(22%) knew about (table 4). Only 98 (41%) mentioned HIV as another major risk at the workplace. None of the participants mentioned viral hemorrhagic fever in spite of the fact that it had been in the news during recent years because of its multiple outbreaks resulting in the deaths of several health care workers at tertiary care facilities in the two cities [14].

**Table 4:** Knowledge of health care workers about modes of transmission of hepatitis B & C and preventive measures at first level health facilities in Sindh province Pakistan, 2004

	**MBBS Prescriber**		**Non-MBBS Prescriber**		**Dispenser**		**Housekeeper**		**Overall N = 239**	
**Mode of transmissions**	n = 42	%	n = 22	%	n = 166	%	n = 9	%	N	%

Reuse of contaminated syringe	39	92.9	16	72.7	117	70.5	2	22.2	174	72.8
Unscreened blood	22	52.4	6	27.3	40	24.1	0	0.0	68	28.5
Unprotected sexual intercourse	22	52.4	6	27.3	41	24.7	0	0.0	69	28.9
Reuse of razor	15	35.7	6	27.3	35	21.1	1	11.1	57	23.8
Use of unsterilized medical instruments	11	26.2	2	9.1	16	9.6	0	0.0	29	12.1
Needle stick or by injury with sharp object	15	35.7	4	18.2	20	12.0	0	0.0	39	16.3
Exposure to body fluids	13	31.0	1	4.5	7	4.2	0	0.0	21	8.8
**Precautions for prevention**										
Use new syringe for each patient	13	31.0	4	18.2	13	7.8	0	0.0	47	19.7
Screen blood before transfusion	2	4.8	0	0.0	3	1.8	0	0.0	5	2.1
Sterilize instrument	3	7.1	0	0.0	6	3.6	0	0.0	9	3.8
Careful to needle	2	4.8	1	4.5	5	3.0	0	0.0	8	3.3
Cleaner work environment (no spillovers, clean dry counters etc)	0	0.0	0	0.0	3	1.8	0	0.0	3	1.3
Appropriate disposal of syringes	3	7.1	1	4.5	9	5.4	0	0.0	13	5.4
Prevention of exposure to body fluids and blood	1	2.4	0	0.0	5	3.0	0	0.0	6	2.5
Watch for unexpected patients movement	5	11.9	0	0.0	6	3.6	1	11.1	12	5.0
General vigilance during work	2	4.8	2	9.1	15	9.0	0	0.0	19	7.9
Avoid unnecessary injections	1	2.4	0	0.0	1	0.6	0	0.0	2	0.8
Don't recapping needle after use	1	2.4	0	0.0	0	0.0	0	0.0	1	0.4
Break needle after use	4	9.5	0	0.0	4	2.4	0	0.0	8	3.3

### Knowledge about measures to prevent blood borne pathogens at workplace

This was an open ended question in which we requested at least three measures that can be used as precautions to prevent exposure to blood borne pathogens at clinics. Mean precaution scores were 1.49 (0.73) Range 0–4. Overall knowledge for most of the items was very low. Use of new syringes was the most highly known factor which 13 (31%) of physicians, 13 (7.8%) of dispensers, and 4 (18.2%) of non-MBBS prescribers mentioned. Few physicians, 3 (7.1%), and even fewer dispensers 9(5.4%) and non-MBBS prescribers 1(4.5%) mentioned appropriate disposal of syringes as a measure to prevent NSIs. Not recapping was mentioned by only one (2.4%) physician. None of the other workers mentioned this point.

Mean score of overall knowledge computed from 11 items regarding mode of transmission and 12 items regarding precautions was 5.30 (2.57), range: 2.0–13.0.

### Relationship between mode of transmission knowledge and risk perception

Mean (SD) risk perception score was 3.43 (1.09) with a median of 4.0. Assessment of relationship between modes of transmission knowledge score and risk perception revealed a weak positive significant correlation (Pearson correlation (ρ) = 0.197, *P *= 0.002).

### Use of universal precautions

Mean (SD) practice score computed from 4 items (three measured on 0–3 and one 0–1), was 2.68 (2.09), with a median of 3 and range: 0–10. Mean of individual items such as wearing gloves and wearing gown during procedures with likelihood of blood or body fluid splashes and not recapping needles after use, was 0.99, 0.36 and 0.91 respectively (table 5).

**Table 5:** Practices of health care workers for protection from occupational exposure at first level care facilities in Sindh province of Pakistan 2004

**Items**	**MBBS Prescriber**		**Non-MBBS Prescriber**		**Dispenser**		**Housekeeper**		**Overall**	
**Completed HBV vaccination**	n	%	n	%	n	%	n	%	n	%

No	7	16.7	14	63.6	96	57.8	2	22.2	119	49.8
Yes	35	83.3	8	36.4	70	42.2	7	77.8	120	50.2
**Wear gloves in procedure where possibility of blood/body fluid exposure**										
Never	15	37.5	11	52.4	82	50.3	3	50.0	111	48.3
Occasionally	5	12.5	3	14.3	38	23.3	2	33.3	48	20.9
Most of the times	13	32.5	4	19.0	16	9.8	1	16.7	34	14.8
Always	7	17.5	3	14.3	27	16.6	0	0.0	37	16.1
**Wear gown for procedures where possibility of blood/body fluid splash**										
Never	29	69.0	16	72.7	129	78.2	2	66.7	176	75.9
Occasionally	11	26.2	5	22.7	23	13.9	0	0	39	16.8
Most of the times	1	2.4	1	4.5	5	3.0	0	0	7	3.0
Always	1	2.4	0		8	4.8	1	33.3	10	4.3
**Needle recap after use**										
Always	24	57.1	14	63.6	98	59.4	1	50.0	137	59.3
Most of the times	1	2.4	1	4.5	23	13.9	0	0	25	10.8
Occasionally	2	4.8	3	13.6	16	9.7	0	0	21	9.1
Never	15	35.7	4	18.2	28	17.0	1	50.0	48	20.8

Majority of MBBS prescribers 35(83%) were vaccinated against hepatitis B while this proportion was lower for dispensers 70 (42%) and non-MBBS prescribers 8 (36.4%). Majority 137 (59.3%) of health care workers always recapped needle after use. Only 15 (37%) of the MBBS prescribers reported never using gloves for procedures while more than 50% of other types of workers never used gloves during performing procedures with potential blood or body fluid exposure (Table 5). The correlation between risk perception and practice score was very weak and not significant (Pearson correlation (ρ) = 0.009, *P *= 0.885).

### Predictors of universal precautions practice score

In multiple linear regression model, modes of transmission knowledge score (adjβ: 0.18, 95% CI: 0.06–0.29) and the work experience (adjβ: 0.06 95% CI: 0.02–0.09) were the only significant predictors of universal precautions score. Since age and work experience were highly correlated, we included only work experience in the model. Hence, the practice of universal precautions depends on knowledge about transmission mode and work experience of the health care worker. Final model explained 9.3% variation in the safety precaution score (Table 6). Residual analysis using the assumptions of normality, linearity and constant variance revealed that the model fits well.

**Table 6:** Predictors of universal precautions practice score (wearing gloves, wearing gown, not recapping and vaccination) among health care workers at first level care facilities in Sindh province Pakistan 2004

	**Univariable models**				**Multivariable model **^a^			
**Variables**	**β**	**F**	***P***	**R**^2^	**adβ**	**F**	***P***	**95% CI of β**

**Knowledge mode of transmission score**	0.22	14.39	< 0.001	0.06	0.18	3.05	0.003	0.06–0.29
**Knowledge of precaution score**	0.34	3.29	0.071	0.01				
**Perceived susceptibility of acquiring infection at workplace**	0.02	0.02	0.885	0.00				
**Perceived Severity of disease after NSI**	0.35	11.83	0.001	0.05				
**Age**	0.05	16.29	< 0.001	0.07				
**Years of work experience**	0.07	16.84	< 0001	0.07	0.06	3.39	0.001	0.02–0.09
**Respondent type**		4.00	0.008	0.04				
MBBS Prescriber	1.75	2.33	0.021					
Non-MBBS Prescriber	0.57	0.70	0.486					
Dispenser	0.62	0.89	0.375					
Housekeeper	Ref							
**Professional qualification**		5.59	< 0.001	0.09				
MBBS	1.73	4.51	< 0.001					
Nursing Diploma	0.74	1.47	0.143					
B-Pharmacy/Homeopathy	0.98	3.14	0.002					
Dispenser/lab technician	1.01	1.61	0.108					
No professional education	Ref							

^a ^Adjusted R^2 ^= 0.093, F statistics = 13.37 *P *< 0.001; adβ = adjusted β, NSI = needle stick injury

## Discussion

Our results indicate that knowledge about the mode of transmission of BBPs was low across all classes of providers. The physicians, however, were better informed as compared to other groups. Knowledge about precautions for preventing exposure to BBPs was also very low. Very few health care workers use universal precautions to lower the risk of BBPs at workplace. Out of knowledge about safety precautions, mode of transmission, risk perception and perception about disease severity, the only factor that predicted universal precautions score was knowledge about mode of transmission. Lack of barrier protection and unnecessary manipulation of injection equipment puts HCWs at risk of sharp injuries that have implications for transmission of HBV and HCV in a country where prevalence of these infections is high among general population.

Ideally, physicians are expected to have a good understanding about the risk of BBPs at work place and about the preventive measures for reducing risk. But as this study found, their knowledge was not adequate. In contrast to this, some other studies in Pakistan have reported a higher level of knowledge and awareness about these risks among residents and physicians working at the tertiary care hospitals. A study of orthopedic residents from all over Pakistan attending a conference reported that 93% of the residents knew that HCV could be transmitted through blood transfusion, 88% knew about its transmission through a needle-stick injury and 74% of subjects had been vaccinated for HBV [15]. A study directed at assessing knowledge of BBPs among medical students in Karachi, Pakistan reported that 100% and 92% of the clinical year's students knew that HBV and HCV could be transmitted through syringes and NSI respectively. Majority of the students (87%) knew that wearing gloves and safe disposal of sharp wastes (98%) protects against these infections while only half of the students were aware that needles should not be recapped [16]. In contrast to this, our study reported only 35.7% of the physicians mentioning needle stick injury as a mode of transmission while this proportion was even lower for other health care workers. Several reasons can be cited for a lower level of knowledge of our workers; our study participants had been in the workforce for an average of nine years; they were thus not fresh medical school graduates. Recent graduates had better knowledge as shown in the above study of residents and medical students. This could be due to incorporation of curriculum addressing occupational safety. Secondly; even if there was no change in the medical curriculum, physicians in cities and at tertiary care hospitals get exposed to infection control practices and learn from wide variety of sources available to them while those at first level care facilities in rural areas may not have access to similar information sources. Lastly there could be methodological differences in the assessment of knowledge. In our study, respondents spontaneously mentioned the transmission routes while in the other two studies referred to above, [15,16] (medical students and orthopedic residents), a list was provided to the participants to choose from. Poor knowledge of our participants as compared to medical students and residents suggests a need for continuous refresher courses in infection control. Our study also included other types of HCWs that have not been studied in Pakistan though their risk of exposure is much higher than physicians since they are directly involved with handling of sharp objects.

A study in India assessed HIV related knowledge among nurses, student nurses, doctors and lab workers during 2002 in 7 rural hospitals managed by a single nongovernmental organization on a 12 item scale [17]. The mean knowledge score was 9.5 (range 4–12, SD 1 [71). These 12 items included statements about mode of transmission of BBPs[17]. Study of compliance with universal precautions in the same hospitals in India showed that 67.6% wore gloves when there was possibility of contact with blood, 53.9% wore apron in procedures whenever there was a possibility of blood or other body fluids splashing and 60.2% did not recap needles. Our sample was from first level care facilities in rural areas that also included doctors and nurses/dispensers; however, most of the participants did not have any formal professional qualification. Private clinics were owned by individual practitioners. In our study, 48% had never worn gloves while only 20.9% wore gloves for most of the time to always, 75.9% reported that they had never used aprons in procedures where there was a possibility of blood or body fluid splash and 59.3% always recapped the needle after use. Although, the knowledge scales are not same, qualitatively health care workers in our study are less knowledgeable and their compliance to universal precautions is also poorer compared to the health care workers in rural areas in India. Differences in knowledge and practice of participants in our study and those in India may be due to differences in size and ownership of facilities, better professional qualification, and access to information. However, similar to the Indian study, knowledge score and years of experience were predictors of practice scores in our study. Differences from the Indian study are also substantiated by better knowledge among HCWs in larger facilities in cities in Pakistan [15,16]. Another study of injection practices at FLCFs in urban and rural North India that assessed the knowledge of HBV and HCV of HCWs reported that 87.5% of the prescribers and 52.5 % of providers (dispensers) knew the association of unsafe injection with HBV. However, association between HCV and unsafe injections was known only to 30 % of prescribers and 5 % of providers. This study also reported that each HCW received 10 NSI every year mostly because of manipulation during sterilization in public facilities and reuse in private clinics [6]. In China, in a tertiary care hospital 94% nurses were aware of HBV and NSI association. However, glove use ranged from 3% to 31% in various activities involving blood and body fluid exposure. Recapping of needles was also common; 30% (135) "always" and 28% (123) "often" recapped needles after use [18]. In developed countries, universal precautions use rate even in community hospitals is considerably higher as compared to our setting. In the United States, a study conducted in two privately owned community hospitals in Minneapolis reported that gloves were observed to be used when appropriate 67.2% of the time, followed by goggles (50.7%), masks (16.0%), gowns (15.3%). Needles were recapped in 34.4% of cases[19]. Another study reported a varied compliance rate regarding universal precautions among hospital physicians in United States: glove use: 94%; disposal of sharps: 92%, wearing protective clothing: 55%; not recapping needles: 56% [20]. Summarizing results from these comparisons suggest that size, ownership of facility and qualifications play an important role in prediction of BBPs' risk at workplace in Pakistan.

Noncompliance is determined by a range of factors including lack of knowledge,[21,20] interference with work skills,[22,23] risk perception,[21,23] conflict of interest,[20,21] not wanting to offend patients,[24] lack of equipment [23,25] and time,[22,23] uncomfortable personal protective equipment (PPE).,[22] inconvenience,[25] work stress,[20] and perceiving a weak organizational commitment to safety climate [20,21]. In our setting, lack of knowledge, poor qualifications, absence of a system for prevention of blood borne pathogens and lack of training, equipment and post exposure prophylaxis at health care facilities are major determinants for non-compliance. The BBP prevention system is present in few tertiary care hospitals and none of the first level care facilities [15,26]. First level care facilities in the private sector are completely different from hospitals because of their size, organization, manpower qualifications and training, and available finances. All these factors influence the BBP prevention program at these facilities and raise important pragmatic and ethical questions. If these health care workers are not aware about the risk at their work place, then who is responsible for enhancing their knowledge and making them aware about workplace risk? Furthermore, who will provide them with the supplies for risk reduction? Should supplies and training be provided by the clinic owner, whoever that may be, or should it's cost be shifted to the consumers (patients)? Provider's fee in these clinics in most of the areas is not much. In our previous studies we estimated that on an average, Rs. 83 (1.3US$) are charged and fee is higher for the GPs [7]. At such low fees, clinic owners may not be willing to provide preventive supplies to their workers. In another study in Sindh province, patients coming from a low socio economic status (SES) were more likely to seek care from the public and unqualified practitioners who charge less. Already, there is a dearth of public health facilities. Shifting cost of supplies to consumers will further make health care less accessible for the poor which will adversely affect their health.

There are several solutions to these problems, but none of them is easy or a 'quick fix'. Most of these solutions need to be incorporated into the overall health care reforms. For instance, health insurance for poor may help to pay for increased expenses and will improve their overall health care seeking ability. Injection use in Pakistan is very high and most of these injections are administered with used injection equipment. A program directed at improving safety and cutting down injection overuse will reduce the risk to health care workers substantially.

In the long run, without a program for occupational safety, health system which is not well resourced in the rural areas can lose health care workers to an epidemic of blood borne pathogens. In such a situation, communication and behavior change programs for health worker risk reduction is a common good that demands immediate governmental investment. Government intervention is also important from the perspective that some of these health workers have very low knowledge about the risk they are exposed to at their workplace and so may not seek information and skills for reduction of their exposure to BBPs.

Initial interventions in the form of communication and behavior change should be backed up by a long term solution for regulating the entry of appropriately qualified staff, regular trainings, and a system for prevention of BBPs incorporating training of staff, NSI surveillance and post exposure prophylaxis.

A number of limitations should be considered while interpreting the findings of this study. We were able to recruit fewer than expected physicians and housekeepers. Physicians mostly do not provide injections and hence their risk of BBP exposure is lower. The housekeepers clean and collect waste without protective equipment and hence are at the high risk of BBP exposure. Majority of the times, they are not full-time employees at the clinics and visit the clinics either very early during the day or late in the evenings. Dispensers who provide injections and handle other sharps are also at a high risk of BBP exposure. We were able to recruit 87% of them from visited clinics. Fewer than expected participation by physicians and housekeepers introduces a possibility of selection bias.

Reporting of practices has been known to be affected by social desirability in the direction of better practices [23]. This could have led to an overestimation of use of gloves, gowns, vaccination for HBV. However, the reported rates were still not very encouraging.

## Conclusion

Knowledge of HCWs about the mode of transmission of BBPs and precautions was low across all types of providers. Physicians, however, were better informed. Very few HCWs used universal precautions to lower the risk of BBPs at their workplace. Poor knowledge determined the very low use of universal precautions that can prevent major proportion of exposure to BBPs. Lack of universal precautions use has implications for BBPs transmissions among HCWs, especially when prevalence of these pathogens in the general population is high. Our findings suggest that training of HCWs to increase their knowledge about BBPs and universal precautions could improve their use of universal precautions. Our discussions with health care workers during this study and in a separate training of master trainers from various health care facilities suggest that HCWs are eager to improve their knowledge about use of universal precautions to protect their health. A model to develop locally relevant educational material and to train master trainers from different areas who in turn educate their peers can work at large scale in short run. Program of training master trainers can be institutionalized at local level through district health departments. However in the long run, a framework incorporating training, supplies, surveillance, and post exposure prophylaxis for prevention of BBP exposure at FLCFs is needed.

## Competing interests

The author(s) declare that they have no competing interests.

## Authors' contributions

NZJ conceived the idea, designed the study, did analysis and prepared and revised the manuscript. SC contributed in design and conduct of study. MR, SR contributed in analysis and write-up. BM contributed in manuscript preparation and revisions. All authors read and approved the final version of manuscript.

## Pre-publication history

The pre-publication history for this paper can be accessed here:



## References

[B1] Beltrami EM, Williams IT, Shapiro CN, Chamberland ME (2000). Risk and management of blood-borne infections in health care workers. Clin Microbiol Rev.

[B2] Pruss-Ustun A, Rapiti E, Hutin Y (2005). Estimation of the global burden of disease attributable to contaminated sharps injuries among health-care workers. Am J Ind Med.

[B3] Haiduven DJ, DeMaio TM, Stevens DA (1992). A five-year study of needlestick injuries: significant reduction associated with communication, education, and convenient placement of sharps containers. Infect Control Hosp Epidemiol.

[B4] Khuri-Bulos NA, Toukan A, Mahafzah A, Al Adham M, Faori I, Abu Khader I, Abu Rumeileh ZI (1997). Epidemiology of needlestick and sharp injuries at a university hospital in a developing country: a 3-year prospective study at the Jordan University Hospital, 1993 through 1995. Am J Infect Control.

[B5] Wang FD, Chen YY, Liu CY (2000). Analysis of sharp-edged medical-object injuries at a medical center in Taiwan. Infect Control Hosp Epidemiol.

[B6] Kotwal A, Priya R, Thakur R, Gupta V, Kotwal J, Seth T (2004). Injection practices in a metropolis of North India: perceptions, determinants and issues of safety. Indian J Med Sci.

[B7] Janjua NZ, Akhtar S, Hutin YJ (2005). Injection use in two districts of Pakistan: implications for disease prevention. Int J Qual Health Care.

[B8] Janjua NZ, Khan MI, Usman HR, Azam I, Khalil M, Ahmad K (2006). Pattern of health care utilization and determinants of care-seeking from GPs in two districts of Pakistan. Southeast Asian J Trop Med Public Health.

[B9] Janjua NZ (2003). Injection practices and sharp waste disposal by general practitioners of Murree, Pakistan. J Pak Med Assoc.

[B10] Hutin Y, Hauri A, Chiarello L, Catlin M, Stilwell B, Ghebrehiwet T, Garner J (2003). Best infection control practices for intradermal, subcutaneous, and intramuscular needle injections. Bull World Health Organ.

[B11] Linnemann CC, Cannon C, DeRonde M, Lanphear B (1991). Effect of educational programs, rigid sharps containers, and universal precautions on reported needlestick injuries in healthcare workers. Infect Control Hosp Epidemiol.

[B12] Glanz K, Marcus Lewis F, Rimer BK (2005). Theory at a Glance: A guide for health promotion practice.

[B13] Janz NK, Becker MH (1984). The Health Belief Model: a decade later. Health Educ Q.

[B14] Athar MN, Khalid MA, Ahmad AM, Bashir N, Baqai HZ, Ahmad M, Balouch AH, Bashir K (2005). Crimean-Congo hemorrhagic fever outbreak in Rawalpindi, Pakistan, February 2002: contact tracing and risk assessment. Am J Trop Med Hyg.

[B15] Rana JS, Khan AR, Halem AA, Khan FN, Gul A, Sarwari AR (2000). Hepatitis C: Knowledge, Attitudes and Practices among orthopedic trainee surgeons in Pakistan. Annals of Saudi Medicine.

[B16] Anjum Q, Siddiqui H, Ahmed Y, Rizvi SR, Usman Y (2005). Knowledge of students regarding hepatitis and HIV/AIDS of a private medical university in Karachi. J Pak Med Assoc.

[B17] Kermode M (2004). Unsafe injections in low-income country health settings: need for injection safety promotion to prevent the spread of blood-borne viruses. Health Promot Int.

[B18] Phipps W, Honghong W, Min Y, Burgess J, Pellico L, Watkins CW, Guoping H, Williams A (2002). Risk of medical sharps injuries among Chinese nurses. Am J Infect Control.

[B19] Henry K, Campbell S, Collier P, Williams CO (1994). Compliance with universal precautions and needle handling and disposal practices among emergency department staff at two community hospitals. Am J Infect Control.

[B20] Michalsen A, Delclos GL, Felknor SA, Davidson AL, Johnson PC, Vesley D, Murphy LR, Kelen GD, Gershon RR (1997). Compliance with universal precautions among physicians. J Occup Environ Med.

[B21] Gershon RRM, Vlahov D, Felknor SA, Vesley D, Johnson PC, Delcios GL, Murphy LR (1995). Compliance with universal precautions among health care workers at three regional hospitals. American Journal of Infection Control.

[B22] Kelen GD, DiGiovanna TA, Celentano DD, Kalainov D, Bisson L, Junkins E, Stein A, Lofy L, Scott CR, Sivertson KT (1990). Adherence to Universal (barrier) Precautions during interventions on critically ill and injured emergency department patients. J Acquir Immune Defic Syndr.

[B23] Henry K, Campbell S, Maki M (1992). A comparison of observed and self-reported compliance with universal precautions among emergency department personnel at a Minnesota public teaching hospital: implications for assessing infection control programs. Ann Emerg Med.

[B24] Ramsey PW, McConnell P, Palmer BH, Glenn LL (1996). Nurses' compliance with universal precautions before and after implementation of OSHA regulations. Clin Nurse Spec.

[B25] Nelsing S, Nielsen TL, Nielsen JO (1997). Noncompliance with universal precautions and the associated risk of mucocutaneous blood exposure among Danish physicians. Infect Control Hosp Epidemiol.

[B26] Hamid SS, Farooqui B, Rizvi Q, Sultana T, Siddiqui AA (1999). Risk of transmission and features of hepatitis C after needlestick injuries. Infect Control Hosp Epidemiol.

